# The Status of the Trained Nurse

**Published:** 1920-06-05

**Authors:** 


*To the Editor of* The Hospital.

### The Status of the Trained Nurse.

*To the Editor of* The Hospital.

Sir,—I quote from an article entitled "The Future Health Services" in The Hospital of May 29. "It has already been pointed out that the Primary Centre would, according to the views of the Council, provide the patient with food, nursing, and all equipment for efficient treatment, but not with medical attendance." Surely now is the time, when the scheme for so big a matter as the systematised health service for England is under construction but not completed, for the College of Nursing to speak on behalf of the nursing profession, which will necessarily be so intimately affected by whatever is brought forward in Dr. Addison's Bill. Why should "nursing" be, as it were, "thrown in" along with board, lodging, and equipment? This is hardly the way to raise the status of the trained nurse in the eyes of the general public. People usually value in proportion to what they pay. Surely in due proportion the nurse's skilled services should be recognised by fees, paid by the patient to the hospital (Primary or Secondary Centre) for her ministrations—(incidentally the hospital could then afford to pay her a higher salary)—equally as the higher fees paid by the patient to the doctor in attendance.—Yours truly,

"A College Nurse."

[Our correspondent should read the paragraph on this subject on p. 257 of the present issue.—Ed. T.H.]

